# Synthesis of a chitosan nanoparticle suspension and its protective effects against enamel demineralization after an *in vitro* cariogenic challenge

**DOI:** 10.1590/1678-7757-2021-0120

**Published:** 2021-09-28

**Authors:** Taís Chaves MAGALHÃES, Natália Moreira TEIXEIRA, Renata Sobreira FRANÇA, Ângelo Márcio Leite DENADAI, Rogério Lacerda dos SANTOS, Hugo Lemes CARLO, Eliseu Aldrighi MUNCHOW, Fabíola Galbiatti de CARVALHO

**Affiliations:** 1 Universidade Federal de Juiz de Fora Universidade Federal de Juiz de Fora Campus Governador Valadares Departamento de Odontologia Governador ValadaresMinas Gerais Brasil Universidade Federal de Juiz de Fora, Campus Governador Valadares, Departamento de Odontologia, Governador Valadares, Minas Gerais, Brasil.; 2 Universidade Federal da Paraíba Universidade Federal da Paraíba Departamento de Odontologia Restauradora ParaíbaJoão Pessoa Brasil Universidade Federal da Paraíba, Departamento de Odontologia Restauradora, Paraíba, João Pessoa, Brasil.; 3 Universidade Federal de Juiz de Fora Universidade Federal de Juiz de Fora Campus Governador Valadares Departamento de Farmácia Governador ValadaresMinas Gerais Brasil Universidade Federal de Juiz de Fora, Campus Governador Valadares, Departamento de Farmácia, Governador Valadares, Minas Gerais, Brasil.

**Keywords:** Chitosan, Nanoparticles, Dental caries, Dental enamel, Hardness

## Abstract

**Objective:**

Our study aims to synthesize, characterize, and determine the effects of a ChNPs suspension on human enamel after cariogenic challenge via pH-cycling.

**Methodology:**

ChNPs were synthesized by ion gelation and characterized by Transmission Electron Microscopy (TEM) and Dynamic Light Scattering. Forty enamel blocks were divided into four groups (n=10/group): (i) ChNPs suspension; (ii) chitosan solution; (iii) 0.05% sodium fluoride (NaF) solution; and (iv) distilled water. Specimens were exposed to cariogenic challenge by cycling in demineralization solution (3 h) and then remineralized (21h) for 7 days. Before each demineralization cycle, the corresponding solutions were passively applied for 90 s. After 7 days, specimens were examined for surface roughness (Ra) and Knoop hardness (KHN) before and after the cariogenic challenge; % KHN change (variation between initial and final hardness), and surface topography by an optical profilometer. The data were analyzed by repeated-measures ANOVA, One-way ANOVA, and Tukey tests (α=0.05).

**Results:**

TEM images showed small spherical particles with diameter and zeta potential values of 79.3 nm and +47.9 mV, respectively. After the challenge, all groups showed an increase in Ra and a decrease in KHN values. Optical profilometry indicated that ChNPs- and NaF-treated specimens showed uneven roughness interspersed with smooth areas and the lowest %KHN values.

**Conclusion:**

The ChNPs suspension was successfully synthesized and minimized human enamel demineralization after a cariogenic challenge, showing an interesting potential for use as an oral formulation for caries prevention.

## Introduction

Dental caries is caused by an imbalance between the presence of undisrupted cariogenic biofilms and the intake of fermentable carbohydrates over time.^1,2^ Frequent toothbrushing with a fluoridated toothpaste is considered the main control mechanism for caries prevention.^2-4^ However, children experiencing poor caries management due to a high frequency of exposure to fermentable carbohydrates, ineffective biofilm removal, or a decrease in salivary flow should use fluoridated mouthwashes as an auxiliary control method.^4,5^

Fluoride prevents caries development through physicochemical mechanisms that minimize tooth demineralization and promote remineralization.^6^ The constant contact of fluoride molecules with the mineral structure of the teeth reduces enamel solubility during the carious process.^5^ Yet, the literature have shown that children, especially those under 6 years of age, may swallow fluoridated toothpaste or mouthwash, which could lead to the onset of dental fluorosis.^5^ This has encouraged the development of alternative natural agents with antimicrobial activity and remineralization effects for the prevention and control of dental caries in susceptible patients. Natural polymers, such as chitosan, have been tested in oral applications due to their antimicrobial properties^7-9^ and role in enamel remineralization.^10-12^

Chitosan is obtained from chitin, which is a linear biopolymer with ß- (1-4) - linked 2-acetamido-2-deoxy-d-glucopyranose and 2-amino-2-deoxy-d-glucopyranose units extracted from the exoskeleton of crustaceans.^9^ A cation derived from the amine group (NH_2_) is amine-protonated at low pH (NH_3_^+^), interacting with negatively charged components, such as proteins, anionic polysaccharides, and phospholipids,^7,8^ fungal and bacterial membranes,^7-9^ as well as with the negatively charged demineralized enamel surface.^12^ After the dissolution of chitosan, the acidic pH can open gaps among the surface layer crystals.^11^ Thus, chitosan may interact with the enamel surface and form a physical barrier and the mineral content loss is minimized by organic acids.^10-12^However, the actual mechanisms by which chitosan can interact with the demineralized enamel remain unclear.

Currently, chitosan is one of the biopolymers most widely used for the synthesis of nanoparticles due to its bioavailability, biocompatibility, biodegradability, and non-toxic characteristics.^13^ Due to their nanometric dimensions, chitosan nanoparticles (ChNPs) have higher absorption and adhesion capacity and greater ability to penetrate the oral biofilm when compared with the chitosan solution.^14,15^ However, the protective effects of a ChNPs suspension against enamel demineralization following an acidogenic simulation remain undetermined.

The ChNPs suspension can be incorporated into a mouthwash as a controlled drug delivery system.^16^ ChNPs formation via the ionic gelation method by a combination of cationic chitosan with anionic compounds, such as sodium tripolyphosphate (TPP), results in hydrophobic precipitates (HNPs) as gel-like nanoparticles.^9^ These structures are stabilized by Lewis acid-base three-dimensional cross-linked interactions between anionic (P-O^-^) and cationic (NH_3_^+^) groups, leading them to the compound precipitates from an aqueous solution.^17^ This nanoparticle suspension can act as a controlled drug delivery system based on ionic dissociation during dilution.^17^ Moreover, it also has mucoadhesion properties and forms a physical barrier,^11,12^ from which the active compounds can be slowly and controlled released into the oral epithelium (alveolar mucosa, cheek, gingiva) and enamel surface.

In our study, we synthesized a ChNPs suspension through ionic gelation, and tested its effects on the enamel surface after cariogenic challenge, simulating the application of a mouthwash. We characterized ChNPs using Dynamic Light Scattering (DLS), Zeta potential (ZP), and Transmission Electron Microscopy (TEM). With an optical profilometer, we assessed the topography and roughness of the enamel surface after treatment and changes in the enamel hardness. The null hypothesis tested was that treatment with the ChNPs suspension simulating a mouthwash application did not change the enamel topography, roughness, and hardness after a cariogenic challenge.

## Methodology

### Synthesis and characterization of the ChNPs suspension

Low molecular weight chitosan (107kDa, 75-85% degree of deacetylation) (Sigma-Aldrich, St. Louis, USA) at 5 mg/mL was dissolved in 1% acetic acid (w/v) (Química Moderna, Barueri, São Paulo, Brazil) and stirred for 24 h at room temperature. The final volume of the chitosan solution was 10 mL. The solution was filtered with 5.0 μm and 0.8 μm membrane filters. The ChNPs suspension was synthesized through the ionic gelation method with the addition of sodium tripolyphosphate (TPP). TPP aqueous solution (Sigma-Aldrich, St. Louis, USA) at 2.4 mg/mL was prepared separately and 3 mL was added to the 10 mL chitosan solution under agitation at 6000 rpm at room temperature using a continuous infusion pump at the rate of 60 mL/h. The final concentration of the ChNPs suspension was 4.4 mg/mL using the chemical formula of mixing solutions. The pH of the suspension was adjusted to 5.5 with the addition of NaOH to match the pH value of some commercial mouthwashes.^18,19^ The pH of the suspension was monitored for 7 days, and the presence of precipitation was also evaluated by visual analysis during the same period.

ChNPs were examined by TEM (Morgagni G20 – FEI, Hillsboro, Oregon, USA) and DLS. For the TEM analysis, a 50 µL aliquot of the ChNPs suspension was dripped in a Holey Carbon grid and remained for 24 h in a desiccator. The formation and morphology of the nanoparticles were observed at 100 kV accelerating voltage. A Zeta Sizer Nano ZS light scattering photometer (Malvern Panalytical, Malvern, UK) was used to measure the hydrodynamic diameter (Dh) of the nanoparticles. Immediately after the synthesis, the ChNPs suspension was inserted into a specific square polyethylene cuvette for particle size measurement. The samples were examined in monochromatic light (10 mW He-Ne laser, 632.4 nm wavelength) and the scattered light intensity was measured at a 90° angle. Dh values were measured 5 times independently, with a mean of 30 counts, at 25°C after 30 s of equilibration time. ZP measurements were obtained in a zeta potential meter (Zeta Sizer Nano ZS, Malvern, UK) by inserting aliquots of the ChNPs suspension into the disposable folded capillary cell of the device (DPS1060).

### Specimen preparation, application of agents, and cariogenic challenge

The sample size was estimated on a pilot study. Considering a standard deviation of 27.0 and a minimal intergroup difference of 50.0 to detect the hardness values of the enamel, a sample of 4 enamel slabs was required to provide 95% statistical power with an α error of 0.05 in a two-sided paired *t*-test. Since the present study tested an experimental agent (ChNPs) that was not tested following a cariogenic challenge, a sample of 10 slabs was used for each group.

Twenty-three healthy third molars were obtained with the approval of the Juiz de Fora Research Ethics Committee (protocol no. 64959517.3.0000.5147). The teeth were stored in 0.1% thymol at 4°C and used within 1 month after extraction. In the proximal surfaces of each tooth, a window of 4 mm × 4 mm was designed. Two slabs (one from each proximal surface) were obtained using a low-speed cutting machine with a water-cooled diamond disc (CutMaster, São Paulo, SP Brazil), totaling an enamel area of 16 mm^2^. The slabs were included in acrylic resin (Vipi Flash, Pirassununga, SP, Brazil) and underwent serial polishing under constant irrigation with silicon carbide (400, 600, and 1200 grades) and alumina suspension (1 μm) (Erios Corp., São Paulo, SP, Brazil). The samples were cleaned for 10 min in an ultrasonic bath (Ultrasonic Cleaner, Unique Ind. and Com. Ltda, São Paulo, Brazil). Each slab was divided into two halves of 4 mm × 2 mm, as follows: (i) control side – coated with two layers of nail varnish (Colorama, CEIL Ltda., SP, Brazil); and (ii) experimental side – subjected to the application of agents and cariogenic challenge.

Specimens with Knoop hardness values ranging between 300 and 380^20^ were selected and randomized into four groups (n=10), as follows: Group 1- ChNPs suspension (4.4 mg/mL, pH 5.5); Group 2- Chitosan solution (5 mg/mL, pH 5.5); Group 3- 0.05% sodium fluoride (NaF) solution (0.5 mg/mL, pH 5.8) (Fluor Sol Clear, Dentsply, Petrópolis, RJ, Brazil) - positive control); Group 4 - Distilled water (negative control) - pH 6.0. Six untreated slabs were used to analyze the surface topography (no cariogenic challenge or application of the agents).

Specimens from each group were subjected to a cariogenic challenge through pH-cycling tests. Each slab was individually fixed in a Falcon tube with the aid of an orthodontic wire and kept in a demineralization solution [2.0 mM of Ca(NO_3_)_2,_ 2.0 mM of Na_2_HPO_4,_ and 75.0 mM acetate at pH 4.7] for 3 h (35.5 mL per specimen) at 37°C. Then, the samples were washed in distilled water and immersed in a remineralization solution [1.5 mM Ca(NO_3_)_2_, 0.9 mM Na_2_HPO_4,_ 150.0 mM KCl in 20.0 mM TRIS buffer solution at pH 7.0] for 21 h (17.7 mL per specimen) at 37°C.^10^This cycle was repeated for 7 days. The demineralization and remineralization solutions were changed daily to prevent saturation/depletion and the accumulation of enamel dissolution agents. To simulate the daily use of mouthwashes, a 20-uL aliquot of the corresponding agents was passively applied with a pipette, without disturbing, for 90 s^10^ before each demineralization cycle. This volume was able to cover the entire enamel without overflow. After the application, all specimens were washed with distilled water.

### Knoop microhardness, surface roughness, and topography

The Knoop Hardness Number (KHN) was obtained using a microhardness tester (HMV-2, Shimadzu, Kyoto, Japan) with a Knoop indenter and a load of 25 g applied for 10 s.^21^ Five indentations with a distance of 100 μm from each other were performed in each slab. The measurements were made in both the control (initial) and experimental sides (after the cariogenic challenge). The percentage of surface hardness change (**%** of KHN change) was estimated using the following formula:^22^

$$\%KHN=100\times \left[\left({KHN}_{\text{post-challenge }}-{KHN}_{\text{initial }}\right)/{KHN}_{\text{initial }}\right]$$

After the cariogenic challenge, the nail varnish was removed from control specimens with the aid of a scalpel blade and the surface was scanned with an optical profilometer (Taylor Hobson - CCI MP, West Chicago, Illinois, USA). To compare the surface topography between treated groups (ChNPs, NaF, Chitosan, and Distilled water) and untreated groups, six specimens were analyzed without cariogenic challenge and application of agents (named Sound Group). The analyses were performed using a 0.008 nm cut-off with a 20× magnification lens at a constant speed of 1 mm/s in XYZ mode (512 × 512-pixel resolution). A Gaussian filter (FALG) according to ISO 16610-61 was used. The length of each scan covered the central slab area with 0.8mm long (X-axis) ×0.8mm wide (Y-axis)  . Three measurements were obtained and averaged to determine the Ra value (in μm) per sample in the TalyMap Lite 7.2 program (64-bit version). The topography of the surface was also examined.

### Statistical analysis

The statistical analysis was conducted using the Jamovi statistical package (Version 1.8, Computer Software, retrieved from www.jamovi.org), considering an α error of 0.05. The data were checked for normal distribution (Shapiro–Wilk test) and equality of variances (Levene’s test), followed by parametric statistical analysis. The hardness and Ra data presented normal distribution (p=0.631 and p=0.645, respectively) and similar variance among the groups (p=0.515 and p=0.524, respectively); hence, repeated-measures analysis of variance (RM ANOVA) and Tukey’s *posthoc* test were applied for: *i)* intragroup comparison of KHN and Ra values before and after the cariogenic challenge; *ii)* comparison of KHN and Ra values among the groups in each experimental period. One-way analysis of variance (ANOVA) and Tukey’s *posthoc* tests were used to analyze the % change in hardness among the groups.

## Results

**Figure 1:**
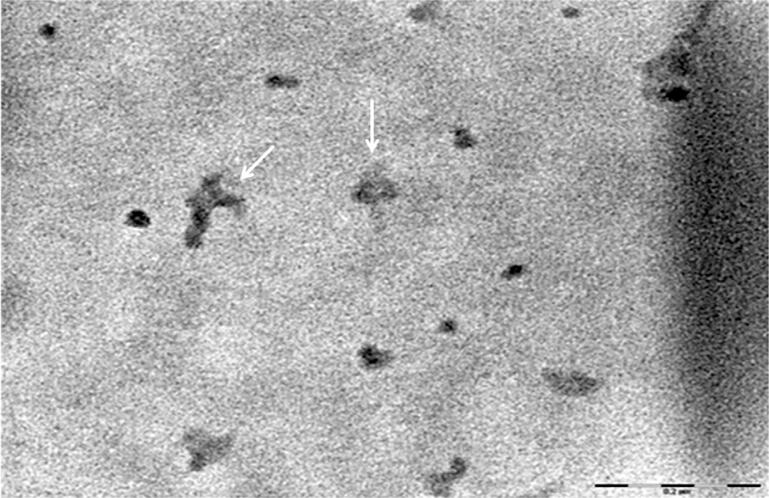
Transmission electron photomicrography of chitosan nanoparticles formation showing dispersed spherical nanoparticles and small areas of aggregation (↓). Reference bar=0.2 µm

The TEM images showed dispersed spherical chitosan nanoparticles and small areas of nanoparticle aggregation (Figure 1). The mean particle size obtained by the DLS method was 79.3±12.5 nm, and the ZP value was + 47.9±6.5 mV. The pH of the ChNPs suspension remained stable for seven days (5.5±0.2). Within this period, no formation of precipitates was found by visual analysis in the suspension.

Table 1 shows the initial and post-challenge microhardness measurements. The cariogenic challenge significantly reduced KHN values in all groups (p<0.001). As expected, initial KHN values did not differ significantly between groups, suggesting that all specimens were matched for hardness (p=0.99). After the cariogenic challenge, specimens treated with the chitosan solution and distilled water showed the lowest KHN values (73.6±11.4 and 69.9±7.8, respectively), with no difference between them (p=1.00). No significant difference was found in KHN values between ChNPs and NaF groups (p=1.00), which had the highest KHN values (108.2±26.4 and 110.3±16.6, respectively). Our findings further indicated that the specimens treated with ChNPs and NaF had the lowest % change in KNH values after the cariogenic challenge (p=0.99), followed by those treated with chitosan and distilled water (p=0.97).

**Table 1:** Knoop hardness values (KHN) of the enamel before and after the application of agents and cariogenic challenge. Values are expressed as mean ± standard deviation

Group	KHN		% of KHN change
(n=10)	KHN _**initial**_	KHN _**post-challenge**_	
Chitosan nanoparticles (4.4 mg/mL)	368.6±15.0^A*,a **^	108.2±26.4^A,b^	70.6±6.8^B^
Chitosan (5.0 mg/ mL)	377.9±19.3^A,a^	73.7±11.4^B,b^	80.5±3.2^A^
Sodium Fluoride (0.5 mg/ mL)	371.8±21.01^A,a^	110.3±16.6^A,b^	70.3±5.0^B^
Distilled water	374.3±19.32^A,a^	69.9±7.8^B,b^	81.3±1.7^A^

*Upper case letters indicate no statistically significant difference among the groups in each experimental period (P>0.05 by the RM ANOVA for KHN and One-way ANOVA for % KHN, followed by post-hoc Tukey tests).

** Values expressed by the same lower-case letters do not have a statistically significant difference in the group before and after cariogenic challenge (P>0.05 by the RM ANOVA and post-hoc Tukey tests).

Table 2 shows the surface roughness (Ra) measurements. No significant difference was observed in Ra values among the groups at baseline (p=0.99) and pos t-challenge (p=1.00). However, when the comparison was made in each group at baseline and post-challenge, Ra values were increased in all groups after the cariogenic challenge (p<0.001), with no difference between them.

**Table 2:** Mean and standard deviation of surface roughness values (Ra) of enamel before and after application of agents and cariogenic challenge. Values are expressed as μm

Group (n=10)	Ra	
	Ra _**initial**_	Ra _**post-challenge**_
Chitosan nanoparticles (4.4 mg/mL)	0.06±0.02^A*,a**^	0.08±0.02^A,b^
Chitosan (5.0 mg/mL)	0.05±0.01^A,a^	0.08±0.02^A,b^
Sodium Fluoride (0.5 mg/mL)	0.05±0.01^A,a^	0.08± 0.01^A,b^
Distilled water	0.05±0.01^A,a^	0.09±0.02^A,b^

* Values expressed by the same upper-case letters do not have a statistically significant difference among groups in each experimental period (P>0.05 by the RM ANOVA for Ra, followed by post-hoc Tukey test).

** Values expressed by the same lower-case letters do not have a statistically significant difference in the group before and after cariogenic challenge (P>0.05 by the RM ANOVA and post-hoc Tukey tests).

**Figure 2:**
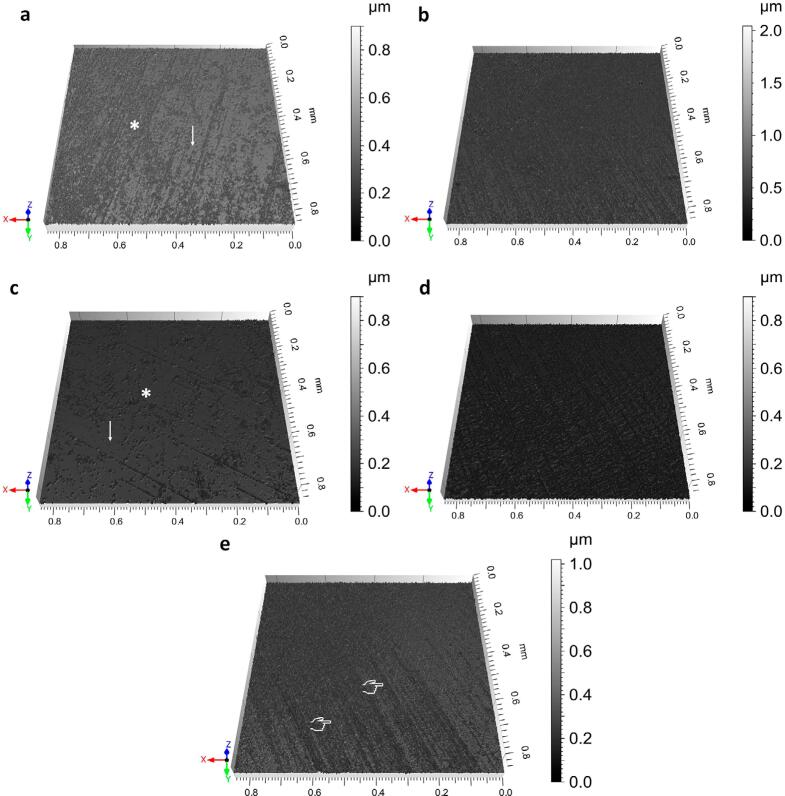
Surface topography of enamel by optical profilometer after application of agents and cariogenic challenge: (a) chitosan nanoparticle suspension; (b) Chitosan; (c) 0.05% NaF and (d) Distilled water (control). Figures a and c show uneven roughness (*) interspersed with smooth enamel areas (↓). Figures b and d depict homogenous and uniform roughness over the entire enamel surface. (e) The sound group shows the presence of some scratches from the specimen polishing (?)

Figure 2 shows the enamel surface topography of sound and treated specimens. The findings show that all groups had specimens with a rough surface. Chitosan and control groups showed homogenous and uniform roughness in all surfaces (Figures 2b and 2d), whereas ChNPs and NaF groups had uneven surface roughness interspersed with smooth enamel areas (Figures 2a and 2c). The sound group showed surface areas with some scratches from the specimen polishing (Figure 2e).

## Discussion

The null hypothesis tested in our study was partially rejected, since ChNPs-treated specimens had their enamel topography and hardness significantly altered after a cariogenic challenge; however, their roughness was not affected.

Chitosan is one of the biopolymers most commonly used for the synthesis of nanoparticles. Several methods can be used in nanoparticle engineering, such as electrospray, emulsification, solvent diffusion, microemulsion, and ionic gelation.^9,13^ In our study, ChNPs were synthesized by ionic gelation using TPP, which is a rapid and easy method for nanoparticle production. The suspension occurs spontaneously through electrostatic interaction between the positively charged amino group of chitosan and the negatively charged polyanions of TPP.^7^ The complex TPP/Chit is stabilized by cross-linked electrostatic interaction between chitosan (NH_3_^+^) and TPP (O^−^ groups), resulting in a three-dimensional entanglement that precipitates from an aqueous solution in the form of gel-like nanoparticles, also named microgels.^9^ TPP has been successfully used for this purpose, because it dismisses the need for harmful organic solvents, heat, or vigorous agitation, rendering the process less expensive and relatively safer.^7^ In our study, we used a stoichiometric proportion of the molar mass (m) of chitosan and TPP to prepare the ChNPs based on a previously published study^9^: $m_{TPP}/m_{\text{chitosan }}=0.18([TPP]/\text{ [chitosan monomer] }=0.09\text{ ) }$ . According to De Carvalho, et al.^9^ (2019), in this proportion, all TPP ions are bound to the chitosan monomer and no free TPP ions are left to act as a protective agent on the enamel during the demineralization process. This is the reason why the TPP solution was not tested in our study as a control group.

The TEM and DLS analyses indicated that ChNPs formation was successful and that there was an interaction between chitosan and TPP molecules. ChNPs had a mean particle size of 79.3±12.5 nm. Several factors can influence the size of nanoparticles, such as the synthesis method, pH of the media, and the concentration and molecular weight of chitosan.^7,23^ These parameters have explained most of the inter-study differences concerning the size of nanoparticles. In our study, we observed the presence of small areas with nanoparticle aggregation (Figure 1). The aggregation or dispersion of ChNPs is governed by the charge interaction. Smaller particles tend to form larger aggregates because of the strong interparticle interaction, as observed in other studies.^7,23^ Positive ZP values (+47.9 mV) are indicative of the presence of free amino groups in chitosan, since the titration with TPP anions was previously shown to reduce the ZP by neutralizing cationic groups.^9^ Moreover, the partial breakdown of tridimensional interchain crosslinking of chitosan by TPP molecules could explain the subsequent formation of slightly charged aggregates.^9^

The pH of the media can also affect relevant physicochemical characteristics of ChNPs suspensions and their stability by changing parameters such as particle hydrophobicity, wettability, and surface homogeneity.^24^ In our study, the pH of the media remained unaltered and, during seven days, no formation of precipitates was found in the suspension, suggesting that the ChNPs suspension was stable.

Surface roughness alterations were detected using profilometer. Their importance on the aggravation of carious lesions was previously discussed by Ando, et al.^25^ The authors showed that, as the enamel undergoes continued demineralization, its surface roughness is progressively increased.^25^ Thus, the authors suggested that the increase in the enamel roughness and the subsequent loss of light reflection can be used to determine the progression of non-cavitated carious lesions.^25^ In clinical practice, these surface properties can be assessed by visual and tactile examinations, which are the most traditional methods to characterize the activity of carious lesions.^25^

In our study, treatment with different agents increased Ra values in all groups, with no significant difference among them (Table 2). No agent was able to prevent the increase in surface roughness, since progressive enamel demineralization was observed. Ra values were obtained from the entire enamel area. In contrast, profilometer images showed that ChNPs and NaF groups had an uneven surface roughness interspersed with smooth enamel areas (Figures 2a and 2c) compared to the uniform roughness observed along the entire chitosan surface and control-treated specimens (Figures 2b and 2d). We hypothesize the application of 0.05% NaF and ChNPs suspension may have protected the enamel surface and diminished demineralization in smooth enamel areas, despite the increased Ra values found. This hypothesis is supported by the microhardness data that will be further discussed herein.

The microhardness test is one of the most used approaches to determine alterations of the enamel surface during the early stages of caries development.^11,21,26^ Although this test cannot point out differences in the mineral content, the surface microhardness assessment is appropriate to investigate the strength of a hard tissue (e.g., enamel) that has a non-homogenous fine microstructure prone to cracking.^21^In our study, we found no significant difference in KHN_initial_ values among the groups (Table 1), which suggests that all specimens were matched for hardness. After the cariogenic challenge, all groups showed a reduction in KHN values (Table 1). This indicates that no agent, not even the 0.05% NaF solution, was able to prevent enamel demineralization. However, ChNPs and 0.05% NaF groups showed lower % change in KHN and higher KHN values compared to those of chitosan and distilled water groups (Table 1). Collectively, these findings indicate that ChNPs and 0.05% NaF successfully strengthened the enamel structure, minimizing the demineralization process and preventing further topographical alterations (Figures 2a and 2c).

The anti-caries effect of mouthwashes containing 0.05% NaF is well known and this effect can result from two major mechanisms of action of fluoride, namely: 1) inhibition of acid-induced demineralization, which is beneficial as fluoridated enamel is more acid-resistant than native enamel; and 2) enhancement of the remineralization of partially demineralized enamel during the early stages of caries development in the presence of calcium and phosphate ions from saliva*.*^27^The anti-caries effects of 0.05% NaF mouthwashes, applied twice daily, were investigated *in vitro.* The data showed that the formulations could prevent enamel demineralization and enhance its remineralization.^28,29^ Consistent with these findings, our study showed that 0.05% NaF can also minimize the enamel demineralization when applied once a day. This is supported by Parkinson, et al.^27^(2018), who demonstrated *in situ* that a 0.05% NaF mouthwash (once daily) had anti-caries efficacy and provided additional benefits when associated with a conventional fluoride dentifrice. However, as suggested by Parkinson et al.^27^ (2018), comparative studies (e.g., day × night use, once × twice-daily use, intermittent use, and fluoride concentrations) are needed to provide evidence-based clinical recommendations.

The effect of a chitosan solution on enamel demineralization was previously investigated through pH-cycling and microhardness tests.^10,12^ Studies showed that the chitosan solution can interact with the tooth surface and form thereon a physical barrier against the penetration of acids. Moreover, chitosan molecules can reduce enamel demineralization by carrying mineral ions deeper into the carious lesions.^10^ According to the literature, only when a chitosan-bioglass complex was used, subsurface mineral deposition was observed, resulting in remineralization of early carious lesions in artificial enamel.^12^

In our study, the chitosan solution (5 mg/mL) could not prevent the reduction of enamel hardness after the cariogenic challenge. Only the ChNPs suspension could minimize the KHN reduction, with no significant difference when compared with the treatment with 0.05% NaF. To the best our knowledge, this is the first study that investigated the effects of ChNPs on enamel properties after pH-cycling. Liu, et al.^14^ (2007) evaluated the adhesion of chitosan nanoparticles contained in a toothpaste but did not use the pH-cycling method. These authors found that ChNPs have a better absorption and adhesion capacity than that of the chitosan solution. The mechanism of action of ChNPs on enamel lesions is still unclear; however, we hypothesize that chitosan nanoparticles are small enough to penetrate the surface layer into the remaining voids of the carious lesions and bond onto the walls of demineralized prisms in a coordinated bond reaction.^30^ The bond can occur by the chemical reaction between hydroxyapatite (metal ions, as Ca^2^) and chitosan (amino groups).^30^

Importantly, besides the beneficial effects of ChNPs on the physical properties of demineralized enamel, the ChNPs suspension can be incorporated into a mouthwash formulation to act as a controlled drug release system in the oral cavity.^16^ChNPs exhibit mucoadhesion to the oral epithelium via mucins, which is an intercellular substance. Mucins are made up of basic units (400–500 kDa) able to join together to form an extended three-dimensional network.^16^ Chitosan has been reported to bind via ionic interactions between primary amino functional groups (NH_3_^+^) and the sialic acid and sulphonic acid substructures of mucins, which are negatively charged.^16^ Unlike the high-molecular-height chitosan solution (pure form), the ChNPs suspension can penetrate the mucin network,^16^ open the tight junctions between the cells, and pass through the mucosal cells.^31^

In short, our study showed that the newly synthesized ChNPs suspension can minimize the enamel demineralization after an acidic challenge. Chitosan is relatively safe due to its biodegradable and biocompatible properties, although ChNPs were reported to be potentially cytotoxic in zebrafish embryos.^13^ Despite these shortcomings, ChNPs are considered promising materials for biomedical applications, including their use as an oral formulation for caries prevention. This preliminary study has some important limitations to consider, namely: i) cross-sectional microhardness testing was not performed; it might reflect indirectly the inorganic content of the enamel substrate after cariogenic challenge and estimate the lesion depth; ii) the penetrability of the ChNPs suspension into the enamel and the mechanism(s) by which chitosan nanoparticles can penetrate the demineralized enamel was not examined; iii) further research should test the use of the ChNPs suspension twice a day, which is consistent with previous studies testing 0.05% NaF mouthwashes. Further *in vitro* studies are needed to determine the cytotoxicity of the ChNPs suspension against oral cells and their antimicrobial effects against multispecies biofilm.

## Conclusions

Our study showed that chitosan nanoparticles were successfully synthesized by the ionic gelation method, and they minimized human enamel demineralization after a cariogenic challenge, showing an interesting potential for use as an oral formulation for caries prevention. Also, ChNPs suspension showed a protective effect on the tooth surface by strengthening the enamel structure and minimizing its demineralization after an *in vitro* cariogenic challenge.
